# Disparate survival of late-stage male oropharyngeal cancer in Appalachia

**DOI:** 10.1038/s41598-020-68380-w

**Published:** 2020-07-15

**Authors:** Brenen W. Papenberg, Jessica L. Allen, Steven M. Markwell, Erik T. Interval, Phillip A. Montague, Christopher J. Johnson, Scott A. Weed

**Affiliations:** 1 Department of Biochemistry, Program in Cancer Cell Biology, West Virginia University Cancer Institute, West Virginia University, P.O. Box 9300, Morgantown, WV 26506 USA; 2 Department of Otolaryngology, Head and Neck Surgery, West Virginia University, Morgantown, WV 26506 USA; 3 Cancer Data Registry of Idaho, Boise, ID 83701 USA

**Keywords:** Cancer epidemiology, Head and neck cancer

## Abstract

The United States Appalachian region harbors a higher cancer burden than the rest of the nation, with disparate incidence of head and neck squamous cell carcinomas (HNSCC), including oral cavity and pharynx (OC/P) cancers. Whether elevated HNSCC incidence generates survival disparities within Appalachia is unknown. To address this, HNSCC survival data for 259,737 tumors from the North American Association for Central Cancer Registries 2007–2013 cohort were evaluated, with age-adjusted relative survival (RS) calculated based on staging, race, sex, and Appalachian residence. Tobacco use, a primary HNSCC risk factor, was evaluated through the Behavioral Risk Factor Surveillance System from Appalachian states. Decreased OC/P RS was found in stage IV Appalachian white males within a subset of states. The survival disparity was confined to human papillomavirus (HPV)-associated oropharyngeal cancers, specifically the oropharynx subsite. This correlated with significantly higher smoking and male smokeless tobacco use in most Appalachian disparity states. Lower survival of Appalachian males with advanced-stage HPV-associated oropharyngeal cancers suggests pervasive tobacco consumption likely generates more aggressive tumors at HPV-associated oropharynx subsites than national averages. Comprehensive tobacco and HPV status should therefore be evaluated prior to considering treatment de-intensification regimens for HPV-associated oropharyngeal cancers in populations with high tobacco consumption.

## Introduction

HNSCC involves the epithelium of the oral cavity, pharynx and larynx. OC/P cancers are a major HNSCC subset, with nearly 11,000 deaths predicted in the US in 2020^1^. Risk factors include tobacco and alcohol use, and high-risk human papillomavirus (HPV) infection^2,3^. OC/P cancers are subdivided into HPV-associated oropharynx (HPV-associated) and non-HPV-associated oral cavity, hypopharynx, and nasopharynx (non-HPV-associated) cancers^4^. These designations are supported by studies indicating that non-HPV-associated cancers are primarily tobacco/alcohol induced, whereas HPV-associated cancers are predominantly caused by HPV infection^5^. Furthermore, HPV-negative or non-HPV-associated cancers consistently have poorer outcomes than HPV-positive or HPV-associated cancers, segregating these cancers as distinct diseases with differential clinical management^6,7^. While national incidence of non-HPV-associated cancers is decreasing due to tobacco cessation, HPV-associated cancers are increasing due to rising infection rates^8^. Regarding race, blacks with HNSCC present with more advanced disease, are older and have worse survival than whites, denoting a racial disparity^9–11^. Increased screening for HPV coupled with subsite analysis indicates that blacks with HNSCC have less HPV-positive cancer than whites, explaining differences in survival^12,13^. Consistent with this, HPV-associated cancers continue to increase in white males and in rural areas^4,14,15^.

The Appalachian region encompasses 205,000 square miles across 420 counties in 13 contiguous states^16^. Forty-two percent of Appalachia is rural, with poverty rates in many counties 1.5 times above the national mean^16^. The region is over 80% white, with several states having higher percentages of oral and other HPV-associated cancers, and higher smoking and smokeless tobacco use^17–19^. Aggregate risk factors experienced by Appalachian residents contribute to a high allostatic load capable of generating cancer disparities in a region with low ethnic diversity^20^. Incidence disparities within Appalachia are well recognized^21^ with rates higher for HPV- and tobacco-related malignancies^22–24^. Elevated risk factor exposure has been suggested as the prime reason for disparate incidence in Appalachian male HNSCC^24,25^. However, due to gaps in data availability and small patient numbers at the county level, details on how increased incidence impacts HNSCC survival in Appalachia have not been rigorously evaluated^25^. Here we have evaluated the RS of all HNSCC in the majority of Appalachian states from 2007–2013 and have identified an outcome disparity in white males with oropharynx cancers that corresponds with elevated smoking and smokeless tobacco use in the region.

## Materials and methods

### HNSCC cohort and relative survival data

All methods were carried out in accordance with relevant guidelines and regulations. Survival data were generated by NAACCR and provided in Surveillance, Epidemiology, and End Results (SEER)*Stat approved under NAACCR IRB protocol 16–14, where all patient data was obtained following informed consent. Patients were diagnosed from 2007–2013 with tumors of International Classification of Diseases for Oncology (ICD-O)-3 histology types 8,050–8,084, 8,120–8,131 (squamous and transitional epithelium). Selecting all primary tumors matching selection criteria, the cohort includes tumors of the oral cavity (n = 55,620/259,737, 21.4%) including ICD-O-3 site groups of tongue (C02.0–02.3, 02.9), floor of mouth (C04.0–04.9), and gum and other mouth (C03.0–03.9, C05.0, C05.8–05.9, C06.0–06.9); oropharynx (n = 81,170/259,737, 31.3%) including ICD-O-3 site groups of tongue (C01.9, C02.4, C02.8), gum and other mouth (C05.1–05.2), tonsil (C09.0–09.9), oropharynx (C10.0–10.9), and “other oral cavity and pharynx” (C14.0–14.8); “other pharynx” (n = 17,731/259,737, 6.8%) including ICD-O-3 site groups of nasopharynx (C11.0–11.9) and hypopharynx (C12.9–13.9); and larynx (n = 60,296/259,737, 23.2%) including ICD-O-3 site group larynx (C32.0–32.9). Remaining tumors are additional primaries of miscellaneous ICD-O-3 site groups (n = 44,916/259,737, 17.3%). The cohort includes variables denoting whether or not patients were Appalachian county residents at the time of diagnosis, and county economic status from the Appalachian Regional Commission (ARC)^26^. Appalachian states with survival data fit for use through the reporting period included New York, Pennsylvania, West Virginia, Kentucky, North Carolina, South Carolina, Georgia, and Alabama^27^. The majority of cases were white (n = 221,939/259,737, 85.4%), stage IV (n = 84,253/259,737, 32.4%), and male (n = 193,647/259,737, 74.6%). Cases were evaluated using the Ederer II method and the “U.S. by race (W, B, AIAN, API) and Canada 1995–2012, Ages 0–99, State-county (modeled by varied state-county-ses)” life table. Included cases were microscopically confirmed for malignant behavior and followed at monthly intervals. Excluded cases were those that were diagnosed solely via death certificate or autopsy, and alive cases with no survival time. Age-adjusted RS, defined as net survival measure representing cancer survival in the absence of other causes of death^28^, was calculated using SEER*Stat for each patient stratification, with z-tests for significance testing between each group using an alpha level of 0.05. Cumulative (C)RS was used to compare groups with 5 years of available RS data and the maximum available follow-up RS was used to compare groups that failed to reach 5 years of follow-up.

### Mapping

Maps were generated using 2014 cartographic boundary shapefiles from the United States Census Bureau^29^ and Quantum Geographic Information System (QGIS) 3.2.2 software^30^. Appalachian state-Appalachian county refers to a county within Appalachia. Appalachian state-non-Appalachian county refers to a county outside of the Appalachian region, but within a state containing Appalachian counties. Non-Appalachian states lack Appalachian counties.

### TCGA cohort and associated clinical data

Data from The Cancer Genome Atlas (TCGA) were obtained from the Broad Genomic Data Analysis Center (GDAC) Firehose 2016_01_28 TCGA-HNSC cohort consisting of 529 patients with tumors of the oral cavity (n = 320/529, 60.5%), pharynx (n = 92/529, 17.4%), and larynx (n = 117/529, 22.1%). Clinical data and patient characteristics were retrieved from the level 4 TCGA clinical data file All_CDEs.txt and cBioPortal^31,32^. OC/P patients were stratified by HPV-associated or non-HPV-associated subsite defined by the Centers for Disease Control (CDC)^33^ and by confirmed HPV status. All patients were evaluated for HPV in the All_CDEs.txt file, denoted by variable “hpv_status”. Kaplan–Meier *P* values were calculated using Mantel-Cox log-rank test and were validated by an independent biostatistician.

### Appalachian tobacco use data and analysis

Data for 2016 current smoker and smokeless tobacco user frequency, weighted frequency, prevalence, and confidence intervals for Appalachian and non-Appalachian counties were calculated and provided by BRFSS coordinators from Appalachian states. Current smoker status was calculated using CDC BRFSS Tobacco Use Question 1 and 2 from the 2016 questionnaire. Current smokeless status was calculated using CDC BRFSS Tobacco Use Question 3. Non-Appalachian state tobacco use data were acquired from CDC BRFSS^19^. Statistical significance was determined within states and between regions using G-tests for independence without Yates’ correction using BRFSS frequency (N) values and an alpha value of 0.05. BRFSS recommends caution interpreting results with less than 50 respondents. Bonferroni correction was used where applicable to reduce type I error. Due to changes in sample composition and weighting methodology in 2011, data from years after 2011 cannot be directly compared to previous years^34^.

## Results

### Identification of a survival disparity in white Appalachian males with stage IV oral cavity and pharyngeal cancer

**Figure 1:**
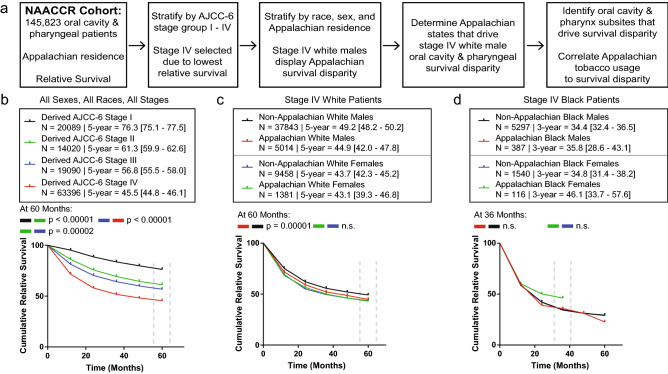
Identification of a survival disparity in white Appalachian male stage IV OC/P cancer. (**a**) flow diagram of procedures used to evaluate Appalachian-specific survival data. Boxes contain rationale and steps involved in the sequential stratification and cohort analysis for OC/P cancer. (**b**) Survival analysis of Appalachian OC/P cancer stratified by clinical stage. CRS values are plotted for each year after diagnosis, with 5-year (60 month) ratios evaluated across all AJCC-6 stages. (**c**) and (**d**). Survival analysis of white (**c**) and black (**d**) stage IV OC/P cancer stratified by Appalachian residency and sex. CRS values are plotted as in (**b**). Patient N, 5-year CRS with 95% CI and *P* values between significant groups are shown at the top of each graph; n.s., not significant. Black patients were evaluated for significance at 36 months due to lack of complete female survival by 48 months and beyond.

The NAACCR cohort from 2007–2013 in this study contained 145,823 OC/P tumors and 57,805 laryngeal tumors (Table 1, Fig. 1a, Fig. S1). The eight NAACCR-reporting Appalachian states cover 67.6% of all Appalachian counties and 72.3% of the Appalachian population^35^. The OC/P and laryngeal cohorts reflect overall Appalachian demographics, with a higher percentage of white cases and lower percentage of ethnic minorities than non-Appalachia (Table 1). Except for lower CRS in Appalachian OC/P and laryngeal cancers, all other parameters were nearly identical to national averages in each disease (Table 1). The majority of OC/P and laryngeal cancer cases came from distressed or transitional Appalachian counties.

**Table 1:** Appalachian and Non-Appalachian OC/P and laryngeal patient characteristics 2007–2013.

2007–2013 NAACCR Cohort characteristics^a^	Total OC/P and laryngeal n = 214,821 (100%)	Total OC/P n = 154,525 (100%)	Appalachian OC/P n = 16,366 (10.59%)	Non-Appalachian OC/P n = 138,159 (89.41%)	Total laryngeal n = 60,296 (100%)	Appalachian laryngeal n = 7,614 (12.63%)	Non-Appalachian laryngeal n = 52,682 (87.37%)
	Mean ± SD	Mean ± SD	Mean ± SD	Mean ± SD	Mean ± SD	Mean ± SD	Mean ± SD
Age (years)	63.05 ± 12.01	62.35 ± 12.22	61.99 ± 12.05	62.39 ± 12.24	64.83 ± 11.26	63.73 ± 11.10	64.99 ± 11.28
	CRS ± SEM	CRS ± SEM	CRS ± SEM	CRS ± SEM	CRS ± SEM	CRS ± SEM	CRS ± SEM
Relative Survival (%)	56.9 ± 0.2	55.0 ± 0.2	52.7 ± 0.7	55.3 ± 0.2	60.1 ± 0.4	58.1 ± 1.1	60.4 ± 0.4
	n (%)	n (%)	n (%)	n (%)	n (%)	n (%)	n (%)
**Sex**							
Male	160,108 (74.5)	111,900 (72.4)	11,779 (72)	100,121 (72.5)	48,208 (80)	6,021 (79.1)	42,187 (80.1)
Female	54,713 (25.5)	42,625 (27.6)	4,587 (28)	38,038 (27.5)	12,088 (20)	1,593 (20.9)	10,495 (19.9)
**Race**							
White	183,029 (85.2)	132,955 (86)	15,049 (92)	117,906 (85.3)	50,074 (83)	6,892 (90.5)	43,182 (82)
Black	23,385 (10.9)	14,769 (9.6)	1,123 (6.9)	13,646 (9.9)	8,616 (14.3)	661 (8.7)	7,955 (15.1)
Other	8,407 (3.9)	6,801 (4.4)	194 (1.2)	6,607 (4.8)	1,606 (2.7)	61 (0.8)	1,545 (2.9)
**AJCC-6 Stage**							
I	40,356 (18.8)	21,555 (13.9)	2,431 (14.9)	19,124 (13.8)	18,801 (31.2)	2,271 (29.8)	16,530 (31.4)
II	23,202 (10.8)	14,622 (9.5)	1,666 (10.2)	12,956 (9.4)	8,580 (14.2)	1,136 (14.9)	7,444 (14.1)
III	28,895 (13.5)	19,498 (12.6)	2,208 (13.5)	17,290 (12.5)	9,397 (15.6)	1,332 (17.5)	8,065 (15.3)
IV	79,924 (37.2)	64,794 (41.9)	7,105 (43.4)	57,689 (41.8)	15,130 (25.1)	1,968 (25.8)	13,162 (25)
Missing	40,938 (19.1)	33,023 (21.4)	2,868 (17.5)	30,155 (21.8)	7,915 (13.1)	850 (11.2)	7,065 (13.4)
**Primary site**							
Oral Cavity	55,620 (25.9)	55,620 (36)	6,028 (36.8)	49,592 (35.9)	–	–	–
Oropharynx	81,174 (37.8)	81,174 (52.5)	8,551 (52.2)	72,623 (52.6)	–	–	–
Other Pharynx	17,731 (8.3)	17,731 (11.5)	1,787 (10.9)	15,944 (11.5)	–	–	–
Larynx	60,296 (28.1)	–	–	–	60,296 (100)	7,614 (100)	52,682 (100)
**ARC economy FY2017**^b^							
Distressed	1,842 (0.9)	1,112 (0.7)	1,112 (6.8)	–	730 (1.2)	730 (9.6)	–
At-Risk	3,491 (1.6)	2,303 (1.5)	2,303 (14.1)	–	1,188 (2)	1,188 (15.6)	–
Transitional	15,935 (7.4)	11,022 (7.1)	11,022 (67.3)	–	4,913 (8.1)	4,913 (64.5)	–
Competitive	2,402 (1.1)	1,701 (1.1)	1,701 (10.4)	–	701 (1.2)	701 (9.2)	–
Attainment	310 (0.1)	228 (0.1)	228 (1.4)	–	82 (0.1)	82 (1.1)	–
Not Applicable	190,841 (88.8)	138,159 (89.4)	–	138,159 (100)	52,682 (87.4)	–	52,682 (100)

*SD* standard deviation, *SEM* standard error of the mean.

^a^Includes data from Alabama, Alaska, Arizona, California, Colorado, Connecticut, Delaware, Florida, Georgia, Hawaii, Idaho, Illinois, Indiana, Iowa, Kentucky, Louisiana, Maine, Michigan, Mississippi, Montana, Nebraska, New Hampshire, New Jersey, New Mexico, New York, North Carolina, Pennsylvania, Rhode Island, Seattle, South Carolina, Utah, West Virginia, Wisconsin, and Wyoming.

^b^ARC county economic status designation for Appalachian counties^26^ is categorized by distressed (Worst 10% of U.S. Counties), at-risk (worst 10 + to 25% of U.S. counties), transitional (worst 25% to Best 25% of U.S. counties), competitive (best 10 + to 25% of U.S. counties), and attainment (best 10% of U.S. counties) based on National Index value rank which is indicated by 3-year average unemployment rate, per capita market income, and poverty rate.

RS was measured among OC/P and laryngeal cancer patients by Appalachian or non-Appalachian residency at time of diagnosis (Fig. 1a, Fig S1). When stratified by American Joint Committee on Cancer (AJCC)-6 stages I to IV, stage IV patients had the highest patient numbers and lowest CRS for each disease (OC/P; n = 63,396/116,595, CRS = 45.5%; laryngeal; n = 14,963/51,362; CRS = 67.1%), and were selected for further evaluation (Fig. 1b and Fig. S1). OC/P and laryngeal patients were stratified by sex, race, and Appalachian residency (Fig. 1c, 1d; Fig S1). Survival analyses indicated that white Appalachian OC/P males (n = 5,014/53,696; CRS = 44.9%) displayed a significantly lower CRS compared to white non-Appalachian males (Fig. 1c; n = 37,843/53,696; CRS = 49.2%), *P* = 0.00001. In OC/P, there was no significant difference in CRS between white Appalachian females (n = 1,381/53,696; CRS = 43.1%) and white non-Appalachian females (n = 9,458/53,696; CRS = 43.7%) (Fig. 1c) or between black Appalachian males (n = 387/7,342; CRS = 35.8%) and black non-Appalachian males (n = 5,297/7,342; CRS = 34.4%) (Fig. 1d). Black Appalachian females (n = 116/7,342; 3-year RS = 46.1%) failed to reach 5-year follow-up for CRS comparison, but there was no observed significance between black non-Appalachian females at the latest available follow-up year (n = 1,540/7,342; 3-year RS = 34.8%) (Fig. 1d). No significant differences in CRS were observed for Appalachian laryngeal cancers between any sex or race (Fig. S1). White Appalachian males with stage IV OC/P were selected for further study due to their significantly different survival.

### The survival disparity in white Appalachian male stage IV OC/P is specific to Appalachian counties within select Appalachian states

**Figure 2:**
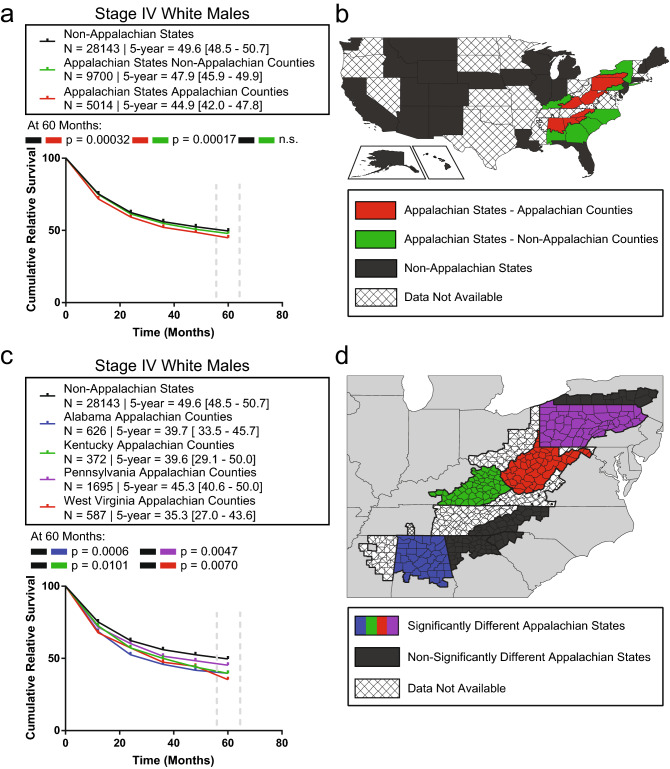
State-level analysis of Appalachian OC/P survival. (**a**) Disparate stage IV white Appalachian male OC/P survival is restricted to Appalachian counties. Plotted CRS values over time are shown for non-Appalachian states (black), non-Appalachian counties within Appalachian states (green) and Appalachian counties within Appalachian states (red). Patient N, 5-year CRS with 95% CI and *P* values at 60 months are shown at the top of the graph; n.s., not significant. (**b**) Mapping of the denoted geographic regions evaluated in (**a**). (**c**) Select Appalachian states with significantly different CRS values contain a survival disparity in OC/P cancer. Appalachian states with significant CRS values are shown plotted over time. Patient N, 5-year CRS with 95% CI and *P* values at 60 months for states with significant survival differences between Appalachian and non-Appalachian counties are shown at the top of the graph. (**d**) Map of Appalachian region displaying states with significantly different stage IV white Appalachian male OC/P CRS. States with significant survival differences between Appalachian and non-Appalachian counties are in black, states with no available data are marked with a cross-hatched pattern.

To elucidate whether the observed stage IV white Appalachian male OC/P survival disparity is specific to Appalachian counties within Appalachian states, patients were stratified by Appalachian or non-Appalachian county and state residency. Appalachian states-Appalachian counties (n = 5,014/42,857; CRS = 44.9%) displayed a significantly lower CRS compared to Appalachian states-non-Appalachian counties (n = 9,700/42,857; CRS = 47.9%), *P* = 0.00017 and non-Appalachian states (n = 28,143/42,857; CRS = 49.6%), *P* = 0.00032 (Fig. 2a and b). This indicates that the survival disparity is specifically due to cancer survival within Appalachia, not to contributions from adjacent non-Appalachian counties within Appalachian state borders. There was no significant difference in CRS between non-Appalachian states and Appalachian states-non-Appalachian counties (Fig. 2a).

To determine if the stage IV white Appalachian male OC/P survival disparity could be identified at the state level, patients within Appalachian counties were stratified by state residency. Significantly lower CRS was observed for Appalachian counties in Alabama (n = 626/31,423; CRS = 39.7%), *P* = 0.0006, Kentucky (n = 372/31,423; CRS = 39.6%), *P* = 0.0101, Pennsylvania (n = 1,695/31,423; CRS = 45.3%), *P* = 0.0047, and West Virginia (n = 587/31,423; CRS = 35.3%), *P* = 0.007 compared to non-Appalachian states (n = 28,143/31,423; CRS = 49.6%) (Fig. 2c,d). All other analyzed Appalachian states did not have a significantly different CRS (Supplementary Figure S2).

### The white Appalachian male stage IV OC/P survival disparity is primarily found in HPV-associated oropharyngeal subsites

**Figure 3:**
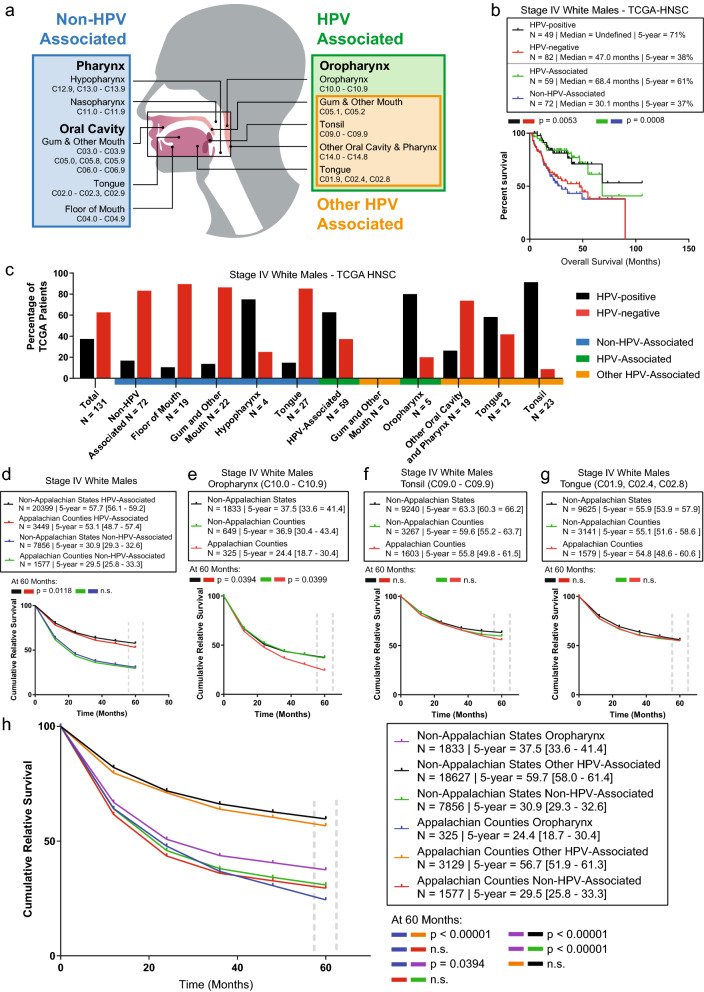
The stage IV white Appalachian male OC/P survival disparity is predominant in HPV-associated oropharyngeal cancers. (**a**) Schematic of the head and neck showing location of non-HPV-associated pharynx and oral cavity (blue), and CDC-defined HPV-associated oropharynx (green) cancers. A subset of the CDC-defined HPV-associated oropharynx is denoted in orange. ICD-O-3 site groups and corresponding site codes for each cancer type are indicated (see “Materials and methods” for detailed description of each site group). (**b**) Overall survival between confirmed and HPV-associated OC/P subtypes. Kaplan–Meier analysis of overall survival of stage IV white males from the TCGA HNSC cohort stratified by HPV status (HPV-positive or HPV-negative) or by ICD-O-3-coded HPV association (HPV-associated and non-HPV-associated). *P* values calculated using Mantel–Cox log-rank test, patient N, median and 5-year overall survival for each group are noted. (**c**) HPV status of OC/P subsites in stage IV white male TCGA patients. Percent of patients with confirmed HPV-negative (red) or HPV-positive (black) disease for each indicated subsite is shown. Denoted subsite groupings are indicated at the bottom as non-HPV-associated (blue), oropharynx (green), or other HPV-associated (orange). (**d**) The stage IV white Appalachian male OC/P survival disparity is present in HPV-associated oropharyngeal cancer. CRS values are plotted over time for stage IV white Appalachian male OC/P patients stratified by Appalachian and non-Appalachian counties and HPV- or non-HPV-associated status. (**e**–**g**) Stage IV white males stratified by HPV-associated oropharynx subsites. Oropharynx (**e**), tonsil (**f**), and tongue (**g**). CRS values are plotted over time with patients stratified by Appalachian counties (red), non-Appalachian counties (green) and non-Appalachian states (black). (**h**) Stage IV white males stratified by non-HPV-associated, oropharynx, other HPV-associated subsites and Appalachian residency. For (**d**–**h**), RS values are plotted over time with patients stratified by Patient N, 5-year CRS with 95% CI and *P* values at 60 months for each group are shown at the top of each respective graph; n.s., not significant.

To determine if the stage IV white Appalachian male OC/P disparity is predominantly present within a specific OC/P subregion, cases were initially divided into non-HPV-associated or HPV-associated cancer subtypes for survival analysis. HPV-associated cancers frequently contain oral mucosa with HPV DNA, and are defined as HPV-associated oropharynx by the CDC^33^. Remaining subsites are not associated with HPV infection and represent non-HPV-associated cancer (Fig. 3a). First, we evaluated the CDC definitions as predictors of HPV infection in HNSCC by using patients in the TCGA cohort, where all patients have known HPV status. Importantly, patients were evaluated for overall survival, since HPV is not a variable collected by NAACCR and thus could not be assessed in the Appalachian cohort. TCGA patients were additionally stratified as HPV-associated and non-HPV-associated using subsite information, then compared to outcomes based on confirmed HPV status. HPV-positive white male stage IV TCGA patients have an undefined median survival with 5-year overall survival of 71%, whereas HPV-negative patients have median survival of 47.0 months and 5-year overall survival of 38% (Fig. 3b). Similarly, HPV-associated cancer patients from the same cohort have median survival of 68.4 months and 5-year overall survival of 61%, while non-HPV-associated patients have lower median survival of 30.1 months and 5-year overall survival of 37%. While the survival trends regarding HPV status are in general agreement between the NAACCR and TCGA cohorts, the difference in 5-year overall survival is greater when comparing HPV-positive (71%) and HPV-associated (61%) patients than between HPV-negative (38%) and non-HPV-associated (37%) patients. This suggests that the CDC non-HPV-associated subsite designation is a better predictor for HPV negativity than the HPV-associated subsite designation is for HPV positivity.

To elucidate the discordance between HPV-positive and HPV-associated patients in explaining the observed Appalachian survival differences, TCGA patients were separated into non-HPV-associated cancers and into the specific HPV-associated oropharynx subsites, with the HPV status determined for each grouping. Using this breakdown, non-HPV-associated cancers are mostly HPV-negative, whilst HPV-associated cancers contain a mixture of HPV-positive and HPV-negative cases (Fig. 3c). These findings potentially explain the discrepancy between HPV-positive and HPV-associated outcomes, and also point to confounding factors such as tobacco/alcohol use or socioeconomic status (SES) that may exist in patient cohorts stratified solely on the basis of HPV-association.

White male stage IV cancer cases in the NAACCR cohort were next stratified by HPV-association and Appalachian residency. Appalachian patients with HPV-associated cancers (n = 3,449/33,281; CRS = 53.1%) displayed a significantly lower CRS compared to HPV-associated patients in non-Appalachian states (n = 20,399/33,281; CRS = 57.7%), *P* = 0.0118 (Fig. 3d). No significant difference was found in CRS between non-HPV-associated patients in Appalachian counties (n = 1,577/33,281; CRS = 29.5%) compared to non-HPV-associated patients in non-Appalachian states (n = 7,856/33,281; CRS = 30.9%). These results suggest that the stage IV white Appalachian male OC/P survival disparity is driven by lower survival of HPV-associated oropharyngeal patients. Appalachian states with the overall stage IV white Appalachian male OC/P survival disparity identified in Fig. 2c were evaluated separately and trended in a similar manner (Supplementary Fig. 3).

White male stage IV patients in the NAACCR cohort were subsequently stratified by each HPV-associated oropharyngeal region to delineate the contributions of each subsite on Appalachian survival. A significant difference in survival was observed between patients with primary oropharynx tumors in Appalachia (n = 325/2,807; CRS = 24.4%) and non-Appalachian counties (n = 649/2,807; CRS = 36.9%), *P* = 0.0399 as well as non-Appalachian states (n = 1,833/2,807; CRS = 37.5%), *P* = 0.0394 (Fig. 3e). No significant differences were observed between patients with primary tonsil tumors in Appalachia (n = 1,603/14,110; CRS = 55.8%) and non-Appalachia states (n = 9,240/14,110; CRS = 63.3%) or counties (n = 3,267/14,110; CRS = 59.6%). Similarly, no significant differences were observed between patients with primary tongue tumors in Appalachia (n = 1,579/14,345; CRS = 54.8%) and non-Appalachian states (n = 9,625/14,345; CRS = 55.9%) or counties (n = 3,141/14,345; CRS = 55.1%) (Fig. 3f,g). Other oropharyngeal subsites lacked sufficient numbers for individual analysis but were combined with tonsil and (base of) tongue to generate an “other HPV-Associated” subset (Fig. 3a).

Regarding survival driven by oropharyngeal tumors, “other HPV-associated” and non-HPV-associated stratification reveals three distinct outcomes in non-Appalachia (Fig. 3h): “Other HPV-associated” (n = 18,627/33,347; CRS = 59.7%) has the best outcome, followed by oropharynx (n = 1,833/33,347; CRS = 37.5%), then non-HPV-associated (n = 7,856/33,347; CRS = 30.9%). However, in Appalachia, only two distinct outcomes are revealed: “Other HPV-associated” (n = 3,129/33,347; CRS = 56.7%) has the best outcome, whereas oropharynx (n = 325/33,347; CRS = 24.4%), has a statistically similar outcome to non-HPV-associated (n = 1,577/33,347; CRS = 29.5%) (Fig. 3h). These results suggest that the observed Appalachian survival disparity is largely driven by lower survival in the oropharynx subregion of HPV-associated stage IV male oropharyngeal patients.

### High Appalachian tobacco use correlates with stage IV white Appalachian male OC/P outcomes

**Figure 4:**
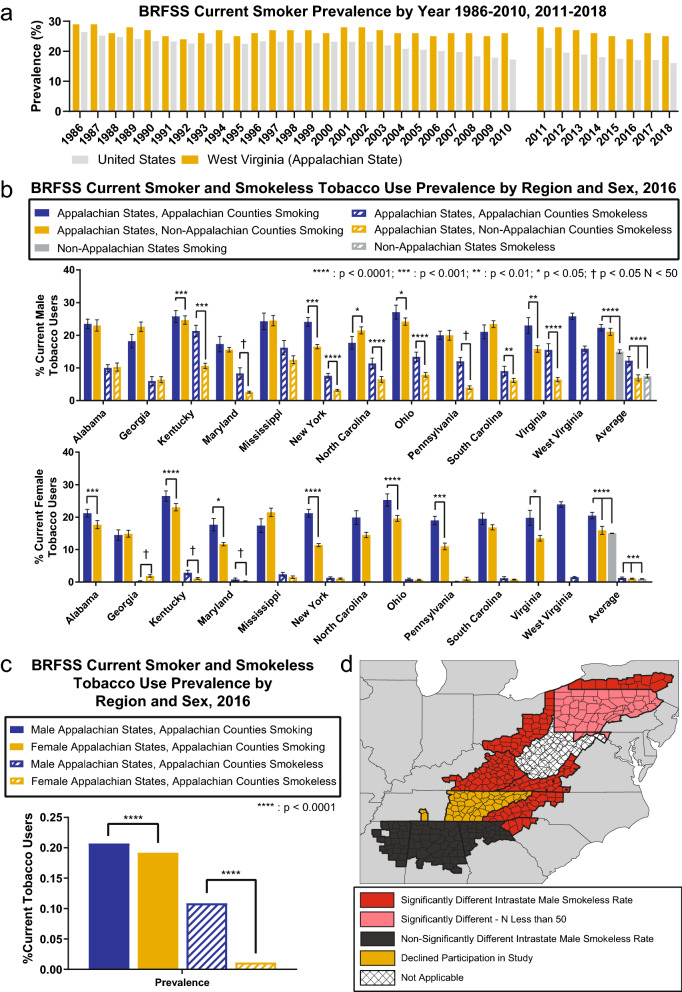
High combined tobacco use in stage IV white Appalachian male OC/P disparity states. (**a**) Overall smoking prevalence rates in West Virginia (as a representation of Appalachia, gold) compared to the United States (grey). Years 2011–2018 cannot be directly compared to earlier years due to differences in weighting methodology across time. (**b**) Elevated tobacco use by Appalachian males (*top*) and females (*bottom*). Data for 2016 smoking weighted prevalence estimates for males in Appalachian states stratified by county status (blue, Appalachian counties; gold, non-Appalachian counties; grey, non-Appalachian states), smoking (solid) or smokeless (cross-hatched) tobacco use with *P* values calculated using G-test for independence without Yates’ correction are shown at *top*. Error bars represent 95% CI. (**c**) Combined Appalachia county smoking (solid) and smokeless (cross-hatched) weighted prevalence estimates with *P* value calculated using G-test for independence without Yates’ correction are shown above significant results. (**d**) Mapping of Appalachian counties smokeless tobacco use. Appalachian counties with significantly higher (red) and non-significantly different (black) smokeless tobacco use are shown compared to non-Appalachian counties within each respective Appalachian state. West Virginia (hatched; non-applicable) lacks non-Appalachian counties for comparison.

The lower survival of stage IV white Appalachian male HPV-associated cancer compared to confirmed HPV-positive national cases implies that risk factors other than HPV are driving poorer survival in the Appalachian cohort (Fig. 3b). Since smoking and smokeless tobacco use are highest in Appalachia^19^, and current smoker prevalence rates have remained higher in West Virginia (a state with all counties within Appalachia) than the nation for several decades^36^ (Fig. 4a), we evaluated tobacco use prevalence rates in Appalachian states from state BRFSS registries stratified by Appalachian residency and sex using data from 2016 as a representative year.

In males, smoking prevalence rates were significantly higher in Appalachian counties compared to non-Appalachian counties, *P* = 4.441e−16 and non-Appalachian states, *P* < 2.2e−16. Specifically, male smoking prevalence rates were significantly higher in Appalachian counties compared to non-Appalachian counties in Kentucky (*P* = 2.80e−04), New York (*P* = 2.55e−04), Ohio (*P* = 0.02965), and Virginia (*P* = 0.008904) (Fig. 4b, top). Male smokeless tobacco prevalence rates were significantly higher in Appalachian counties compared to non-Appalachian counties, *P* < 2.2e−16 and non-Appalachian states, *P* < 2.2e−16. Specifically, male smokeless tobacco prevalence rates were significantly higher in Appalachian counties compared to non-Appalachian counties in Kentucky (*P* < 2.2e−16), New York (*P* = 9.03e−7), North Carolina (*P* = 5.44e−6), Ohio (*P* = 1.87e−12), South Carolina (*P* = 0.00419), and Virginia (*P* = 6.63e−9). Significant differences were also observed in Maryland and Pennsylvania, but these regions had fewer than 50 respondents.

West Virginia contains no non-Appalachian counties for comparison, but the smoking tobacco rate of 25.8% and the smokeless tobacco rate of 15.9% are similar to that of other states with significant differences in the region (Fig. 4b, top). Interestingly, except for Alabama, states with significantly higher male smokeless tobacco use include every state with a significant difference in CRS for stage IV white Appalachian male OC/P and HPV-associated cancers (Fig. 4d).

In females, average smoking prevalence rates were higher in Appalachian counties compared to non-Appalachian counties, *P* < 2.2e−16, and non-Appalachian states, *P* < 2.2e−16 (Fig. 4b, bottom) and were statistically different than that of Appalachian males (20.7% males vs. 19.2% females) (Fig. 4c). Smoking prevalence rates were significantly higher in Appalachian counties compared to non-Appalachian counties in Alabama (*P* = 1.5e−4), Kentucky (*P* = 1.33e−9), Maryland (*P* = 0.02284), New York (*P* = 3.4e−9), Ohio (*P* = 9.38e−5), Pennsylvania (*P* = 1.44e−4), and Virginia (*P* = 0.0362). Female smokeless tobacco prevalence rates were significantly higher in Appalachian counties compared to non-Appalachian counties, *P* = 8.76e−4 and non-Appalachian states, *P* = 2.912e−05, and were significantly different than that of Appalachian males (10.9% males vs. 1.2% females), *P* < 2.2e−16 (Fig. 4c). Significant differences were observed in Kentucky and Maryland, although these regions had fewer than 50 respondents. These data indicate that significantly higher smoking and smokeless tobacco rates within Appalachia correlate with disparate survival of stage IV white Appalachian male OC/P patients in most Appalachian disparity states.

## Discussion

With a majority of population and geographical coverage, our direct, non-exploratory analysis of HNSCC survival from available states representing all ARC-defined Appalachian subregions^16^ identifies stage IV white Appalachian males with OC/P cancer as having lower cancer-related survival compared to non-Appalachian males. Based on available data, this stage- and sex-specific disparity is manifest within the Appalachian areas of four states. The disparity is restricted to the ICD-O-3 defined oropharynx (C10.0–10.9) within the CDC-defined HPV-associated oropharyngeal region. This disparity predominantly occurs in states with significantly higher male smoking and smokeless tobacco use, consistent with high risk-factor exposure in Appalachia known to contribute to increased HNSCC incidence^22,24,25^.

Previous work evaluating Appalachian oral cancer survival using 2004 SEER data identified lower combined male and female survival in 10 states without consideration of stage or race^25^. When stratified by stage, race and sex, our multivariate analysis indicates that the only significant difference in CRS occurs in white Appalachian stage IV male OC/P patients diagnosed under AJCC-6^37^. RS values for stage IV white Appalachian and non-Appalachian male OC/P are higher than that of black males or black females, in agreement with a recognized national survival disparity for black OC/P cancers attributed to lower overall SES and cultural barriers^9,11^. In addition, female white stage IV OC/P patients have a lower CRS than male white stage IV OC/P regardless of Appalachian status, likely reflecting the lower rate of HPV-positive oropharyngeal disease in females^38,39^. Emerging national trends indicate that HPV-associated oropharyngeal cancers occur more frequently in younger, white male patients with limited tobacco use, and within rural areas^4,14,38^. These factors, coupled with blacks and white females having higher percentages of HPV-negative disease^12,13^ are congruent with HPV-positive and/or HPV-associated white male oropharyngeal patients having higher survival at the national level.

OC/P patient populations consist of a mixture of HPV-negative and HPV-positive disease. White Appalachian males have a higher incidence in HPV-positive OC/P cancers, which would be expected to result in increased survival. However, stage IV-matched CRS for white Appalachian males is closer to the lower survival prevalence rates observed for white females than for non-Appalachian white males (Fig. 1c). Furthermore, male Appalachian stage IV HPV-associated oropharynx patients exhibit outcomes similar to non-HPV-associated male stage IV disease within and outside of Appalachia. Of the recognized cancer risk factors endemic to Appalachia, age at diagnosis, travel distance to critical care centers and disproportionate presentation of patients at stage IV have been reported to have no impact on survival in Appalachian subpopulations^40,41^ (Table 1). However, increased smoking and smokeless tobacco use within Appalachia have been linked to low SES and increased HNSCC incidence^42–44^, and thus are presumably factors contributing to the decreased male stage IV oropharyngeal cancer survival.

Our findings also indicate that Appalachian smoking tobacco prevalence rates, represented by West Virginia, have been higher than national averages for several decades (Fig. 4a), and while Appalachian smoking tobacco use is significantly higher in males and females, smokeless tobacco use is primarily higher in males compared to national averages (Fig. 4b). Smokeless tobacco use has been specifically linked to increased cancer risk in OC/P and oropharyngeal subsites^45–48^. Elevated smoking and smokeless tobacco use in most Appalachian states with significantly different CRS corresponds with decreased CRS in white male stage IV OC/P cancers. This has the potential to result in the male patient population garnering a higher percentage of tobacco-induced HPV-negative disease at all oral subsites, including the oropharynx. While the lack of diagnostic HPV detection by p16 staining as a variable in the NAACCR cohort precludes direct analysis of viral status, segregation of white male stage IV OC/P patients by non-HPV-associated OC/P and HPV-associated oropharynx indicates that HPV-associated oropharyngeal cancer is responsible for the Appalachian male stage IV disparity within ICD-O-3 oropharynx codes. The predominant subsites under this delineation are C10.0 (vallecula), C10.1 (anterior surface of epiglottis), C10.2 (lateral wall of oropharynx), C10.3 (posterior wall of pharynx) and C10.4 (branchial cleft). These oropharyngeal regions exclude palatine and lingual tonsils, as well as most other HPV-associated oropharyngeal sites containing the reticular epithelium lining the tonsillar crypts that constitute the primary sites of oropharyngeal HPV infection and neoplasia^49–51^. Higher aggregate tobacco use by Appalachian males may therefore result in more frequent carcinogen-induced malignancy at oropharynx subsites, resulting in a greater percentage of HPV-negative disease in the oropharynx and corresponding worse CRS, similar to that observed in other tobacco-heavy oropharyngeal cohorts^3,52,53^.

The Appalachian-specific OC/P and oropharynx-specific disparities found in this study were diagnosed under the AJCC-6 timeframe, having clinical and pathological staging guidelines independent of HPV status. Restaging of HPV-positive oropharynx in 2018 under AJCC-8 due to the favorable prognosis of HPV-positive oropharyngeal cancers would shift most HPV-positive oropharynx in AJCC-6 to lower clinical stages. Such restaging would have the effect of potentially eliminating or downstaging the Appalachian male disparity described in this report. However, an additional ramification from this work is that male Appalachian or other populations with multi-factorial oropharyngeal tobacco exposure may actually be under-staged using current AJCC-8 guidelines. This is an important consideration, since National Comprehensive Cancer Network (NCCN) guidelines recommend staging and treatment dependent on p16 status^52,54^, where treatment de-escalation of HPV-associated oropharyngeal cancers continues to be evaluated^55–57^. Future efforts towards definitively determining the extent of HPV involvement in Appalachian OC/P and oropharynx through comprehensive p16 staining and PCR^58^ will be required to better clarify the predominant factors underlying oropharynx-driven, stage-based disparities in past and future Appalachian cohorts.

### Limitations and implications

While the current study covers the majority of the Appalachian population and is the most comprehensive study of HNSCC survival of the region to date, lack of available qualified survival data from five Appalachian states prevented complete assessment of survival in the region. The association between heavy aggregate tobacco use and poor male stage IV oropharyngeal survival in states with significantly different CRS suggests that stage IV oropharyngeal patients from states with Appalachian regions containing similar tobacco use patterns may also harbor disparate outcomes. Our findings, in conjunction with the poor survival of HPV-positive oropharynx patients with high smoking histories^59,60^, underscore the need for the comprehensive tobacco history of any HPV-positive oropharynx patient to be considered prior to treatment. In addition to smoking, the significantly higher smokeless tobacco use by the male Appalachian population further increases the risk of tobacco-induced cancer at HPV-associated oropharyngeal sites, leading to oropharynx tumors that are either HPV-negative, or are HPV-positive but exhibit aggressive HPV-negative tumor behavior.

## Conclusions

These findings provide novel and in-depth insight into a specific demographic within a chronically underserved, rural population that is at higher clinical risk for poor OC/P outcome. Persistent high tobacco usage in Appalachia, in spite of increased tobacco cessation efforts, reinforces the need for continued targeted risk awareness. This includes emerging forms of supposed safer nitrosamine-containing products, such as e-cigarettes, that contribute to the cumulative patient tobacco load^61^. Inclusion of patient use of these products as standard registry variables should be considered for improved monitoring of tobacco-related disparities in future populations. The described male oropharynx disparity may also be present in male patients from other regions with heavy smoking, smokeless tobacco^62^ or betel-quid^63^ use, and should be monitored accordingly for similar poor survival and continued cessation intervention policies.

## Supplementary information

Supplementary file1 (DOCX 774 kb)

## Data Availability

The data that support the findings of this study are available upon application and IRB approval from NAACCR, thus these data are not in the public domain. Data are however available from the authors upon reasonable request and with prior approval by NAACCR.
