# Supplementary material for: Disparate survival of late-stage male oropharyngeal cancer in Appalachia
Source: Sci Rep. 2020 Jul 15;10:11612. doi: 10.1038/s41598-020-68380-w (PMC7363863; doi:10.1038/s41598-020-68380-w)
Supplement: Supplementary file 1 — Supplementary file1 (DOCX 774 kb) [file 41598_2020_68380_MOESM1_ESM.docx]

**­­­Disparate Survival of Late-stage Male Oropharyngeal Cancer in Appalachia**

Brenen W. Papenberg^1^, Jessica L. Allen^1^, Steven M. Markwell^1^, Erik T. Interval^2^, Phillip A. Montague^2^, Christopher J. Johnson^3^ and Scott A. Weed^1*^

**Supplemental Materials**
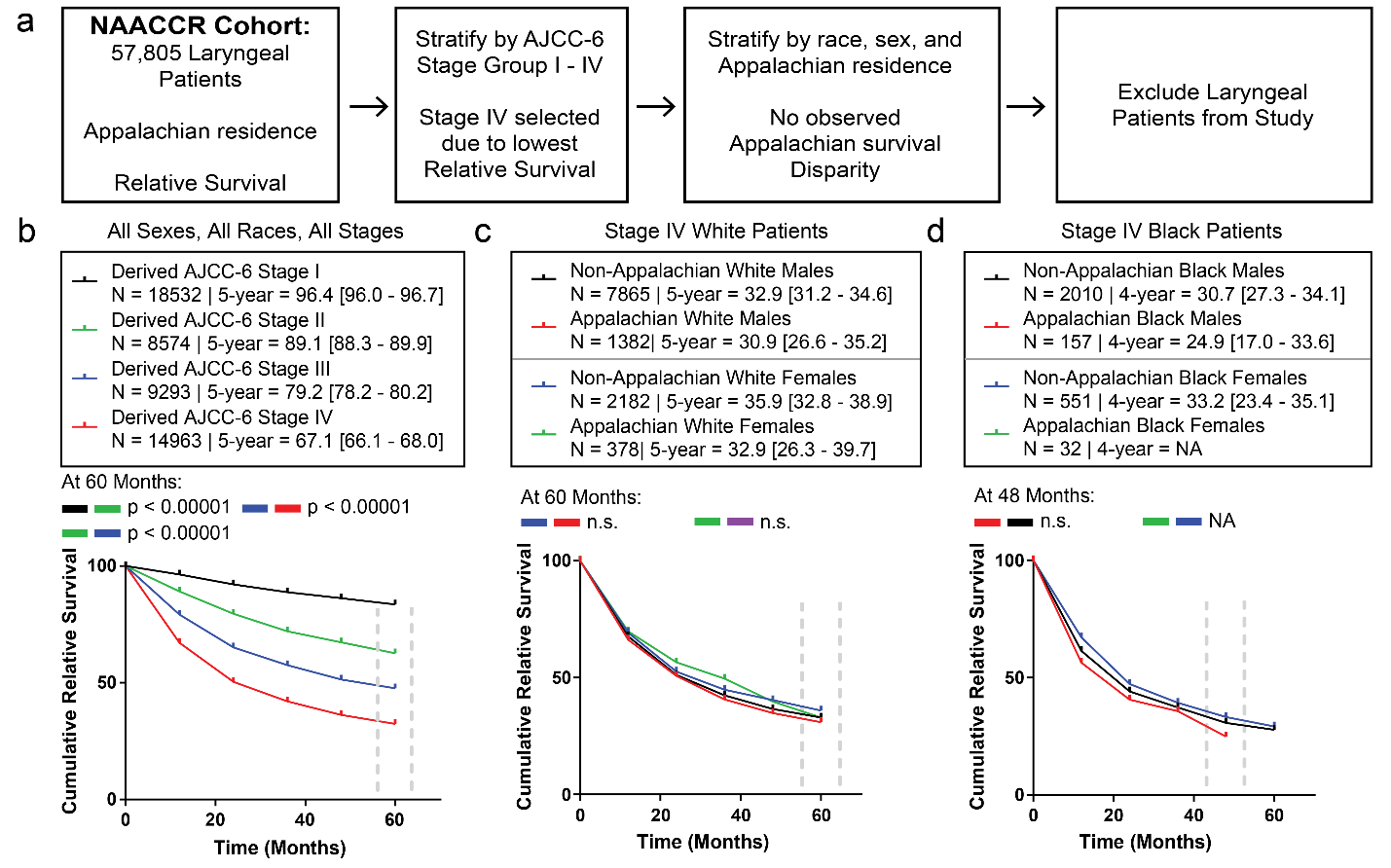
**Supplementary Figure S1. Survival analysis of Appalachian Stage IV laryngeal cancer.** **A.** Flow diagram of procedures used to evaluate Appalachian-specific laryngeal cancer survival data. Boxes contain sequential operational steps utilized in the stratification and cohort analysis, noting no difference in survival within the cohort. **B.** Survival analysis of Appalachian laryngeal cancer stratified by clinical stage. RS values are plotted for each year after diagnosis, with five-year (60 month) ratios evaluated across all AJCC-6 stages. **C** and **D.** Survival analysis of white (**C**) and black (**D**) stage IV laryngeal cancer stratified by Appalachian residency and sex. RS values are plotted as in (**B**). P-values between significant groups are shown at the top of each graph; n.s., not significant; NA, not available. Black patients were evaluated for significance at 48 months due to incomplete male survival at 60 months.


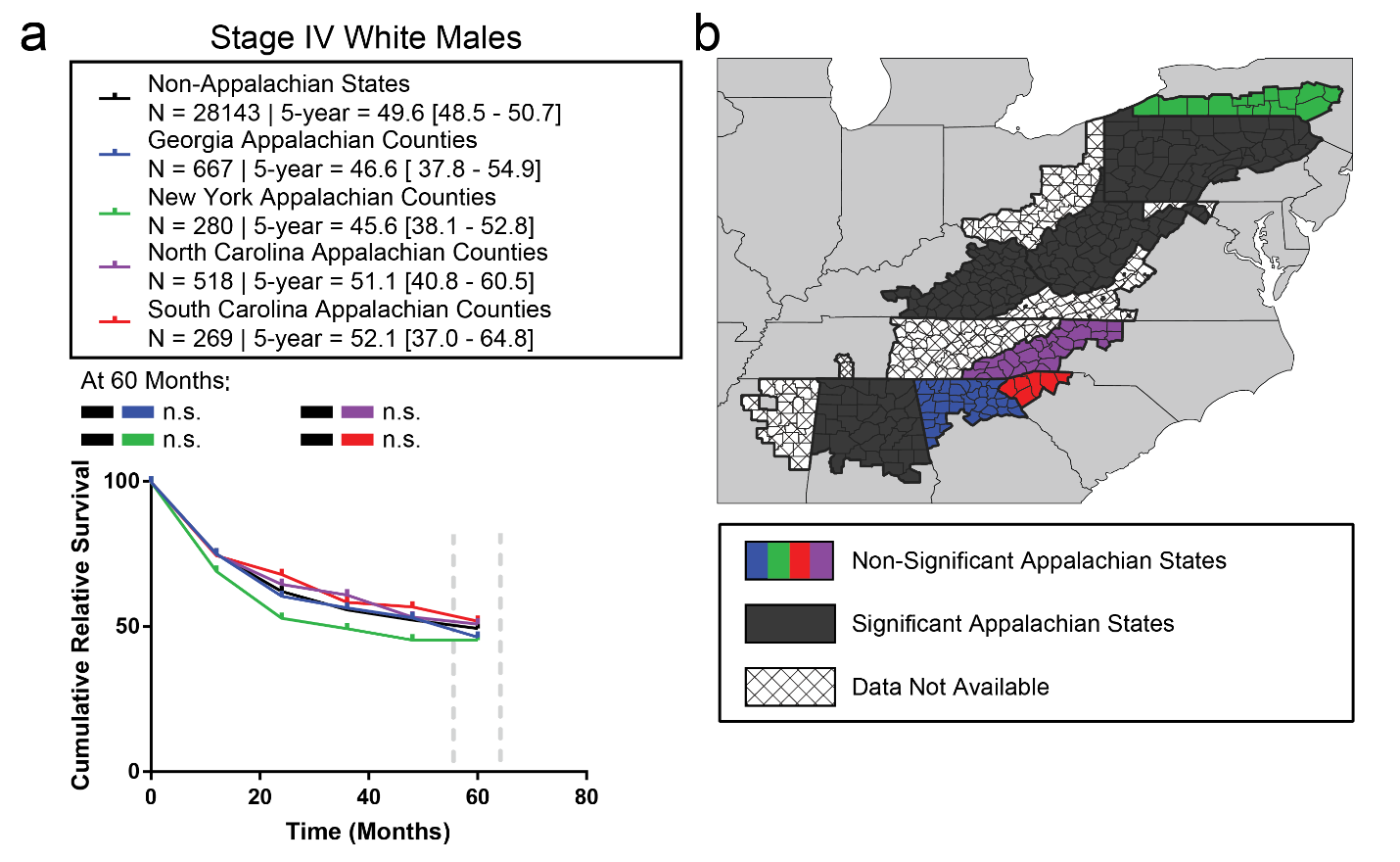


**Supplementary Figure S2. Survival analysis of Appalachian states with non-significant OC/P survival.** **A.** States lacking disparate RS of stage IV white Appalachian male OC/P. Plotted RS values over time are shown for non-Appalachian states (black), and Appalachian states with non-significant (n.s.) survival. Patient N, five-year CRS with 95% CI and P-values at 60 months for each significant Appalachian state are shown at the top of the graph. **B.** Map of Appalachian region displaying states with non-significant stage IV white Appalachian male OC/P survival. Colored states are non-significant, and significant states are in black; states with no available data are marked with a cross-hatched pattern.


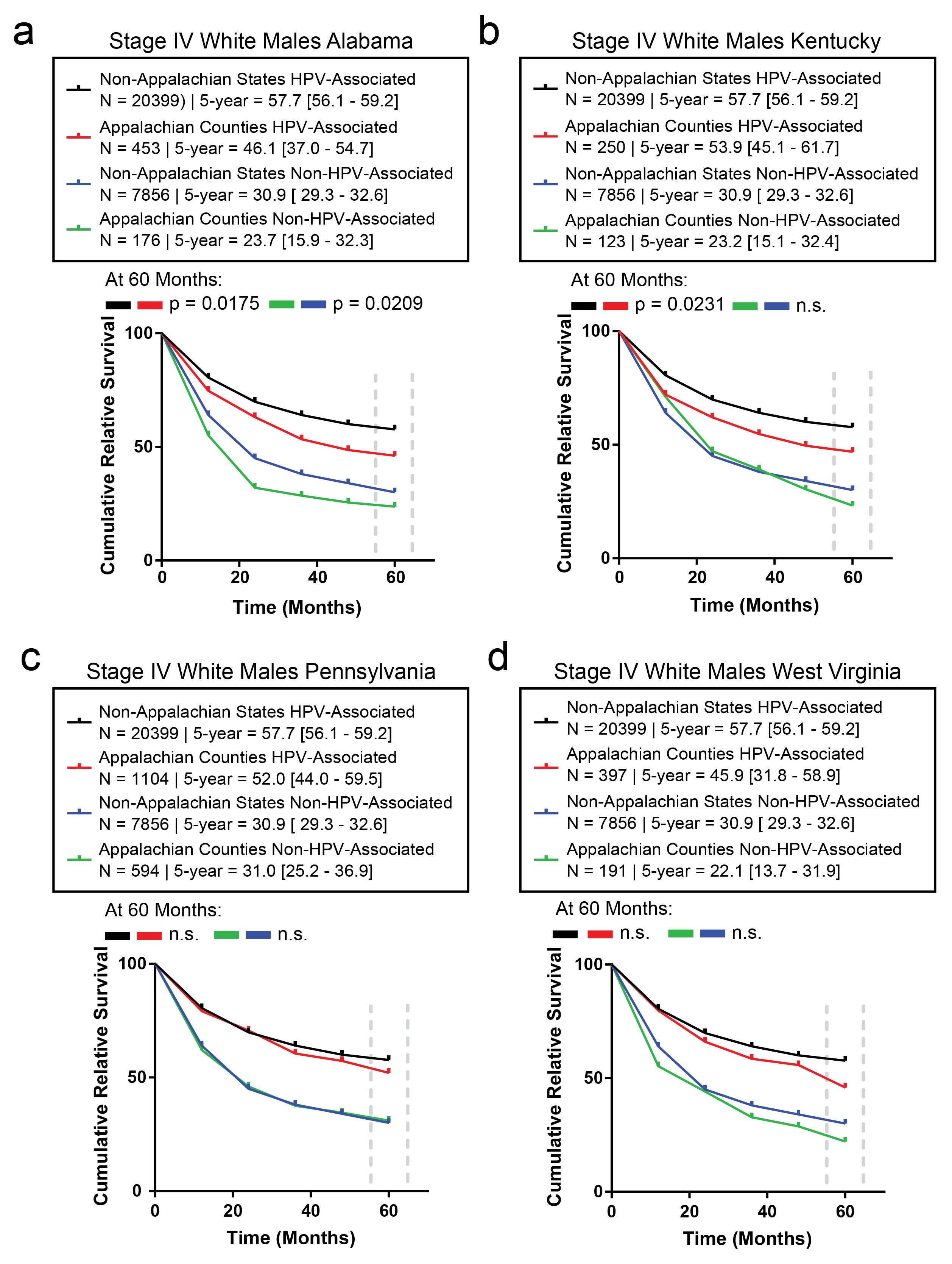


**Supplementary Figure S3. Survival analysis of Appalachian states with non-significant OC/P survival. A-D**. Identification of states with disparate stage IV white Appalachian male HPV-associated oropharyngeal cancer survival. RS values were plotted for patients stratified as in Figure 3D for each OC/P disparity state. **A**, Alabama; **B**, Kentucky; **C**, Pennsylvania; **D**, West Virginia. Patient N, five-year CRS with 95% CI and P-values at 60 months for each Appalachian state are shown at the top of the graph; n.s., not significant.
